# Analysis of the Pomelo Peel Essential Oils at Different Storage Durations Using a Visible and Near-Infrared Spectroscopic on Intact Fruit

**DOI:** 10.3390/foods13152379

**Published:** 2024-07-27

**Authors:** Panmanas Sirisomboon, Jittra Duangchang, Thitima Phanomsophon, Ravipat Lapcharoensuk, Bim Prasad Shrestha, Sumaporn Kasemsamran, Warunee Thanapase, Pimpen Pornchaloempong, Satoru Tsuchikawa

**Affiliations:** 1 Department of Agricultural Engineering, School of Engineering, King Mongkut’s Institute of Technology Ladkrabang, Bangkok 10520, Thailand; panmanas.si@kmitl.ac.th (P.S.); yoki.ami@hotmail.com (J.D.); 2 Office of Administrative Interdisciplinary Program on Agricultural Technology, School of Agricultural Technology, King Mongkut’s Institute of Technology Ladkrabang, Bangkok 10520, Thailand; thitima.ph@kmitl.ac.th; 3 Department of Mechanical Engineering, School of Engineering, Kathmandu University, Dhulikhel P.O. Box 6250, Nepal; 4 Department of Bioengineering, University of Washington, William H. Foege Building 3720, 15th Ave. NE, Seattle, WA 98195-5061, USA; 5 Kasetsart Agricultural and Agro-Industrial Product Improvement Institute (KAPI), Kasetsart University, Bangkok 10600, Thailand; aapspk@ku.ac.th (S.K.); aapwnt@ku.ac.th (W.T.); 6 Department of Food Engineering, School of Engineering, King Mongkut’s Institute of Technology Ladkrabang, Bangkok 10520, Thailand; pimpen.po@kmitl.ac.th; 7 Graduate School of Bioagricultural Sciences, Nagoya University, Nagoya 464-8603, Japan; st3842@agr.nagoya-u.ac.jp

**Keywords:** visible/near-infrared spectroscopy, essential oil, pomelo, partial least squares (PLS)

## Abstract

Pomelo fruit pulp mainly is consumed fresh and with very little processing, and its peels are discarded as biological waste, which can cause the environmental problems. The peels contain several bioactive chemical compounds, especially essential oils (EOs). The content of a specific EO is important for the extraction process in industry and in research units such as breeding research. The explanation of the biosynthesis pathway for EO generation and change was included. The chemical bond vibration affected the prediction of EO constituents was comprehensively explained by regression coefficient plots and x-loading plots. Visible and near-infrared spectroscopy (VIS/NIRS) is a prominent rapid technique used for fruit quality assessment. This research work was focused on evaluating the use of VIS/NIRS to predict the composition of EOs found in the peel of the pomelo fruit (*Citrus maxima* (J. Burm.) Merr. cv Kao Nam Pueng) following storage. The composition of the peel oil was analyzed by gas chromatography–mass spectrometry (GC-MS) at storage durations of 0, 15, 30, 45, 60, 75, 90, 105 and 120 days (at 10 °C and 70% relative humidity). The relationship between the NIR spectral data and the major EO components found in the peel, including nootkatone, geranial, β-phellandrene and limonene, were established using the raw spectral data in conjunction with partial least squares (PLS) regression. Preprocessing of the raw spectra was performed using multiplicative scatter correction (MSC) or second derivative preprocessing. The PLS model of nootkatone with full MSC had the highest correlation coefficient between the predicted and reference values (r = 0.82), with a standard error of prediction (SEP) of 0.11% and bias of 0.01%, while the models of geranial, β-phellandrene and limonene provided too low r values of 0.75, 0.75 and 0.67, respectively. The nootkatone model is only appropriate for use in screening and some other approximate calibrations, though this is the first report of the use of NIR spectroscopy on intact fruit measurement for its peel EO constituents during cold storage.

## 1. Introduction

The negative environmental issues created by agriculture and food wastes are important and critical in this world. But the emerging production of bioactive compounds from waste and plant secondary metabolites is an economic and profitable method of waste management. The essential oils (EOs) extracted from food waste can have a significant role in solving this environment problem.

The pomelo (*Citrus maxima* (J. Burm.) Merr. cv Kao Nam Pueng) is an important fruit for export in Thailand. It is the largest in the citrus fruit family and contains a thick, spongy peel [1]. The pomelo peel contains a number of EO constituents. In some parts of the oriental world, the decoction of the fruit peel provided a medicinal compound that alleviated coughs, ulcers, swellings and epilepsy. It was reported that pomelo EO can activate fat metabolism in the body, which has an anti-obesity effect [2]. The EO has also been used as an effective natural antioxidant [3]. As described by Xie et al. [4], one of the EOs, nootkatone, is a flavorant (FEMA 3166) used in food and tobacco [5] and also used as an antiulcer agent [6] as well as displaying insecticidal activity against *Drosophila melanogaster* [7]. Phellandrene compounds extracted from pomelo peel are used in fragrances [8,9]. Limonene, one of the terpenoids in nature, is the major constituent of EOs and due to its pleasant citrus fragrance, it is commonly used as a flavor in foods and drinks [9,10], for which it is classified in the U.S. Code of Federal Regulation as safe. The tests in animals have proven the effectiveness of limonene against some types of cancer including gastric, mammary, pulmonary adenoma and liver varieties [9,10]. Limonene has also been effective in treating gastroesophageal reflux disorder and occasional heartburn [11].

Pomelo fruit pulp is mainly consumed fresh and with very less processing, and its peels, which is about 30% of the total fruit weight [12], are discarded as biological waste. which can cause environmental problems. The content of a specific EO is important for the extraction process in industry and in research units such as breeding research.

Near-infrared spectroscopy (NIRS) was used in the evaluation of citrus EO components such as limonene, myrcene, α-pinene, β-pinene, sabinene, γ-terpinene to scan the oil itself using the long NIR wavelength (1100–2500 nm). Steuer et al. [13] indicated that the multiple coefficients of determination (R^2^) for the component with an amount of more than 1.5% were generally more than 0.95. The citral EO content in lemongrass EO and lemon EO was evaluated by longwave NIRS, and it was shown for the lemongrass oils, the mean accuracy and bias of citral EO content was ≤1.00% and ≤0.09%, respectively, and for the lemon oils, it was ≤4.28% and ≤−0.71%, respectively; therefore, the developed NIRS protocol was non-destructive, rapid and simple and may prove to accurately evaluate the citral content of lemongrass oils and the approximate citral content of lemon oils [14]. In addition, NIRS was used to determine the EO components in rose-scented geranium EO, and the calibration models produced good R^2^ of > 0.90 for seven major volatile constituents (geraniol, citronellol, geranyl formate, citronellyl formate, linalool, isomenthone and guaia-6,9-diene) and error parameters after external validation was low (<1.0%) [15]. The cross-validation models developed by NIR spectra predicted accurately almost all of the components of African EOs from cinnamon (*Cinnamomum zeylanicum*), clove (*Syzygium aromaticum*), *Cinnamomum camphora* (ravintsara), *Ravensara aromatica* (ravensara) and *Lippia multiflora* with R^2^ ≥ 0.985 and exhibited a low variance (less than 5%) [16]. The EOs obtained from various chemotypes of thyme, oregano and chamomile species were studied by NIRS, where the main EOs components (e.g., carvacrol, thymol, γ-terpinene, α-bisabolol and β-farnesene) models provided R^2^ > 0.97 [17].

The longwave NIRS models have been used in the evaluation of the constituents of fruits and vegetables by scanning the intact fruit for pectin-related constituents, including alcohol-insoluble solids in the fresh weight (AIS in the FW) and the oxalate-soluble pectin content in the AIS (OSP in the AIS) in Japanese pear with correlation coefficient (R) = 0.93, standard error of prediction (SEP) = 0.62 for AIS in the FW and R = 0.95, SEP = 8.48 for OSP in the AIS [18]. The estimation of sucrose, glucose and fructose of the citrus fruits scanned by longwave NIRS provided an R^2^ of 0.996–0.998 [19]. Mango fruits were determined for firmness, total soluble solids (TSS), titratable acidity (TA), pH, β-carotene content and ripening index (RPI) by fruit spectra acquired by longwave NIR spectrometer for developing calibration models, for which the R^2^ of 0.84, 0.85, 0.89, 0.88, 0.86 and 0.90, respectively, were obtained [20].

The VIS/NIR spectroscopy was used, for example, the prediction of TSS and pH of kiwifruit where the transmission spectra of kiwifruit were obtained in the wavelength range from 400 to 1000 nm, and the R for TSS and pH models were 0.93, 0.943 and the root mean square error of prediction (RMSEP) was 0.259 °Brix and 0.076, respectively [21], and the prediction of models based on VIS/NIR in the 500–1050 nm range collected from the intact fruit of banana and mango for estimation of dry matter (DM) and TSS of mesocarp tissue provided R^2^ > 0.75 and a root mean square error of cross-validation (RMSECV) of <0.70% DM for mango; however, the performance of the banana mesocarp DM model was relatively poor, presumably due to the thickness of the peel. For mango, TSS was modeled well only in ripened fruit (RMSECV < 0.60%) and was predicted poorly across ripening stages [22].

However, the VIS/NIRS has been long applied to measure the physicochemical properties of citrus, including pomelo. The ability of Vis/NIRS to non-destructively predict the sweetness and flavor attributes of oranges and grapefruit reported the best prediction models for BrimA (Brix minus acids) of ‘Valencia’ oranges with the coefficient of R^2^ = 0.958 and the TSS:TA ratio (total soluble solids to titratable acid ratio) (R^2^ = 0.958). Good models for predicting the flavor of grapefruit were also attained with less ability due to the thick peel caused a weak spectral signal with TSS (R^2^ = 0.896), followed by BrimA (R^2^ = 0.858) [23]. Shatian pomelo is a traditional Chinese large, thick-peeled fruit, and granulation and water loss are two major internal quality factors that influence its storage quality. Xu et al. [24] showed that the water content of the postharvest pomelo model provided an R^2^ of 0.712 and the detection of granulation degree accuracy of the validation set was 100%. Additionally, for intact oil, the VIS/NIRS technique for the evaluation of acidity and peroxide of olive oils provided an R^2^ of 0.88 and 0.86 for validation sets, respectively [25].

There has been no reported literature regarding the use of NIRS in the evaluation of the EOs in the peel by scanning intact citrus fruit. This is confirmed by the review by Magwaza, et al. [26] in 2012, where no papers on intact fruit scanning for the evaluation of EOs, to our knowledge, existed until now with our work on the determination of EOs in pomelo peels with different maturity [27]. The evaluation of the EO of citrus fruit by traditional methods is resource-consuming in terms of both the time, chemicals, as well as the requirement of expert skills. The use of NIRS for this process is advantageous, as it is relatively simple, rapid and the lack of chemicals needed means the measurement process is environmentally friendly.

The aim of this study was to present a fast and reliable VIS/NIR (600–1100 nm) spectroscopy method for the evaluation of major EO components in the peel of pomelo by scanning intact fruit that was kept in cold storage and a comprehensive discussion of the rationale as to why the VIS/NIRS model was more or less feasible. Only the results for nootkatone, geranial, β-phellandrene and limonene were reported here as it was found that the NIRS method used in this work was not suitable to evaluate other essential oils (β-Pinene, α-Pinene, α-Phellandrene, germacrene).

## 2. Materials and Methods

The dataset used in this study consisted of 135 pomelo fruits that were obtained from Nakornchaisri, Nakornpathom province, located in central Thailand. The 300 bud flowers before blooming were randomly tagged and the harvesting of the 135 fruit was conducted at commercial harvesting time, 210 days after initial flower blooming. Fruit samples were brought to King Mongkut’s Institute of Technology Ladkrabang, Bangkok, Thailand. The pomelo fruits were then waxed (Nature Bright, Israel) for storage. The fruits were stored for differing number of days, 0, 15, 30, 45, 60, 75, 90, 105 and 120 days (10 °C and 70% relative humidity), before analysis using NIRS (15 fruits per date). Due to our experiment being 120 days (~4 months or ~12 weeks) using long storage at 10 °C and 70% relative humidity, we then divided the sampling date to be 15-day intervals to observe the obvious change and to reduce the number of sampling dates (9 sampling dates). The storage of 120 days was followed 12–13 weeks storage at 11 °C (simulated sea transportation to Japan) [28].

### 2.1. VIS/NIR Scanning

**Figure 1:**
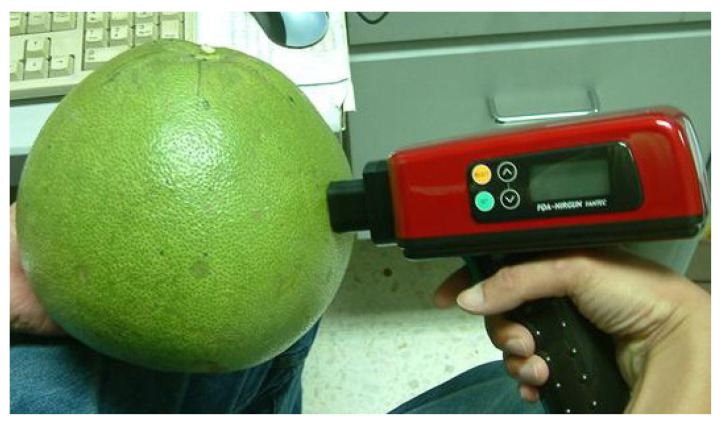
Scanning of pomelo fruit.

Before spectra measurement, the pomelos were removed from storage and kept at 25 °C for 24 h to bring the temperature of pomelos to room temperature. At the beginning of measurement, the Teflon white standard was measured for reference. The NIR diffuse reflection spectra of each pomelo were measured in the range of 600–1100 nm with a resolution of 2 nm and integration time of 20 ms using a FQA NIR Gun (Fantec Inc., Osaka, Japan) as shown in Figure 1. The fruit and the FQA NIR Gun were covered with black cloth to prevent interference from external light. The VIS/NIR diffuse reflection spectra were obtained at 5 positions at the equatorial area 72° apart. At each position, three scans were performed, and an average spectrum (15 scans) was calculated for each fruit.

### 2.2. Reference Analysis

Essential oil of each fruit sample was extracted from the pomelo peel using a cold-pressing method modified from Njoroge, et al. [29]. The pomelo albedo was peeled off by hand using a sharp knife and discarded to obtain the flavedo with its oil glands. The crude EO was collected on ice and centrifuged at 4000× *g* for 15 min at 5 °C. The solution was decanted, dried with anhydrous sodium sulfate for 24 h at 5 °C and filtered to obtain the cold-pressed EO, which was kept at −20 °C prior to analysis. The components of the EO were determined by gas chromatography–mass spectrometry (GC-MS) using a GC (6890N Agilent Technologies, Santa Clara, CA, USA) and MS (5973 Agilent Technologies, USA), fitted with a J&W DBwax 30 m × 0.25 mm column (film thickness: 0.25 µm). Injector and detector temperatures were 250 °C, the oven temperature was programmed to 70 °C (2 min) with an increase of 2 °C per minute to 230 °C (20 min); the carrier gas was helium (He). EO sample of 0.2 µL was injected. The major EO components of peel, including nootkatone, geranial, β-phellandrene and limonene, were evaluated. The mean of the composition percentage at different storage durations were differentiated by Duncan’s multiple range test with confidence level of 95%. There were 2–3 replicates per each fruit sample.

### 2.3. Data Analysis

Data processing and the appropriate chemometric models were performed by the Unscrambler 9.8 software (Camo ASA, Oslo, Norway). In the calibration, the spectra at 663–961 nm were used, as these were without significant noise. A principal component analysis (PCA) technique was applied to check outlier spectra before calibration. Also, some spectra were removed to obtain rectangle-like data distribution for uniformity of prediction accuracy in a range. The dataset randomly separated into a calibration set and a prediction set using a ratio of 5:2, respectively. The randomization was performed by running the sample data in ascending order and the first 5 samples were assigned to calibration set and the following 2 samples were assigned to prediction set and this procedure was continued until every sample was assigned, and the maximum and minimum reference value samples were assigned to calibration set. This is to ensure the same distribution of the calibration set and prediction set to avoid overfitting. NIR models were developed using partial least squares (PLS) regression and the original spectra, or original spectra with either full multiplicative scatter correction (MSC) or second derivative correction. The second derivative correction was obtained by the Savitzky–Goley method with two different derivative segments, either 11 or 21 points, with a polynomial order of two. The number of significant principal components (PCs) for the PLS algorithm was determined using the cross-validation method [30]. The prediction error sum of squares (PRESS) obtained in the cross-validation was calculated each time that a new PC was added to the model. Haaland and Thomas [31] criterion, where the comparison of the PRESS from models (h models) with the model involves the number of PCs yielding the minimum PRESS (h∗ model), was applied for the selection of the optimum number of PCs. The F-statistic is used to make the significance determination with a value of α = 0.25.

The calibration accuracy is described by the correlation coefficient (r) (Equation (1)), standard error of prediction (SEP) (Equation (2)) and bias (Equation (3)) [32].

(1) $$r=\frac{\sum \mathrm{XY}-\left[\frac{\left(\sum X\sum Y\right)}{N}\right]}{\sqrt{\left[\sum X^{2}-\left(\frac{\sum X^{2}}{N}\right)\right]\left[\sum Y^{2}-\left(\frac{\sum Y^{2}}{N}\right)\right]}}$$

(2) $$\mathrm{SEP}=\sqrt{\frac{\sum {(X-Y)}^{2}-\left\{\frac{{\left[\sum (X-Y)\right]}^{2}}{N}\right\}}{N-1}}$$

(3) $$\mathrm{bias}=\frac{\sum (X-Y)}{N}$$

where X is measured data, Y is NIRS predicted data and N is number of observations.

## 3. Results

Table 1 shows the major EO components of pomelo peel (cv Kao Nam Pueng), including nootkatone (0.06–0.63%), geranial (0.27–0.40%), β-phellandrene (4.64–6.32%) and limonene (79.17–84.61%), at storage durations of 0, 15, 30, 45, 60, 75, 90, 105 and 120 days (10 °C and 70% relative humidity).

**Figure 2:**
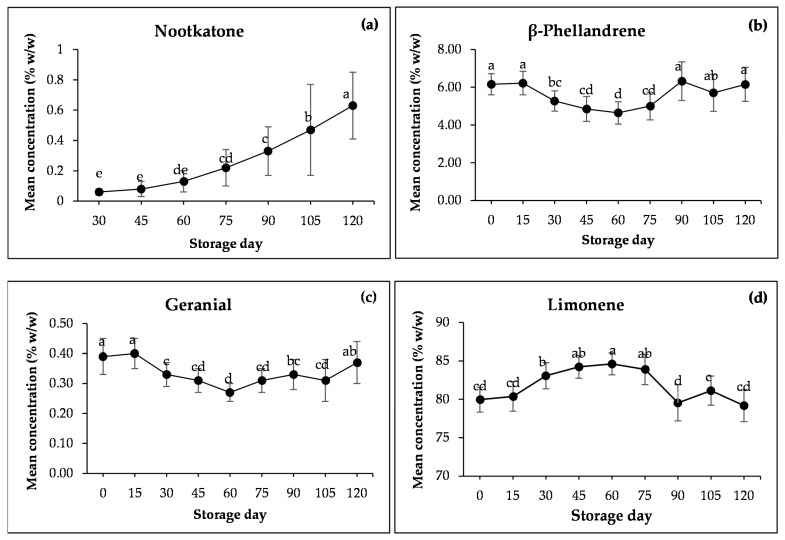
The change in concentrations of (**a**) nootkatone; (**b**) β-phellandrene; (**c**) geranial; (**d**) limonene in pomelo peel during fruit storage. Different letters in each data point represent significant difference at *p* ≤ 0.05 by Duncan’s multiple range tests.

Table 1 and Figure 2 show that the nootkatone contained in the samples at 0 and 15 days of storage was small, and it could not be detected by GC-MS. However, it increased with increasing storage duration. β-phellandrene was constant in the first 15 days and then decreased in amount from 15 days of storage to 60 days and then tended to increase to 90 days and then seemed to be unchanged until 120 days was reached. Geranial displayed the same trend as β-phellandrene with a different concentration. The limonene concentration had the mirror trend in β-phellandrene and geranial, meaning it had the same range of constant amounts but decreased when the amount of β-phellandrene and geranial increased or vice versa from 0 days to 120 days.

During the storage of the pomelo, there was no peel damage, which was the same as the storage of lemon for the duration of the experiment for fruit stored at 10 °C [33]. Liu et al. [34] indicated the change in flavedo during storage, such as the different constituents of carotenoids, might imply their independent EOs synthesis or accumulation in different fruit tissues during storage time. Figure 2 shows the change in concentrations of nootkatone (a), β-phellandrene (b), geranial (c) and limonene (d) in pomelo peel during fruit storage. Compounds, terpenoids, are dominant in the taste, aroma, and pigment profiles of pomelo fruits [34]. These EO constituents were the volatile terpenoids in the flavedo of pomelo. Thus, the fruit tissues might be involved with the regulation mechanism in their precursors’ production, channeling and translocation [34]. Nootkatone is sesquiterpene ketone, β-phellandrene is monoterpene hydrocarbon, geranial is monoterpene aldehyde and limonene is monoterpene hydrocarbon.

Recently, an exchange of intermediates occurring between plastids and cytosol under normal circumstances, is still considered as sesqui- and triterpenoids being synthesized through the cytosolic mevalonic acid (MVA) pathway and mono-, di- and tetraterpenoids through the plastid methylerythritol phosphate (MEP) pathway [34,35,36]. Indeed, the activation of amino acid and fatty acid degradation to generate tricarboxylic acid (TCA) cycle acetyl-CoA (Acetyl-coenzyme A) precursors and thus maintains energy production that leads to the accumulation of specific substrates for volatile formation [37]. Therefore, there were different trends of change in different EOs during storage.

Sesquiterpene ketone nootkatone is known as a characteristic volatile compound present in pomelo peel EOs [25] and is usually used to differentiate pomelo and grapefruit EOs from the other citrus oils. The sesquiterpenoids nootkatone was germacrene-type sesquiterpenoids, which is the product of the MVA pathway. Its relative percentage increases with maturity and storage [25,38]. From Figure 2a, nootkatone was increased from less than 0.06% to 0.63% when the storage time was 4 months. It was reported that nootkatone was formed in vivo from valencene in cell suspension cultures of a kind of lemon (*C. limon* cv. Ponderosa) [39]. Thus, it is possible that nootkatone is synthesized from valencene [40]. Sawamura et al. [40] indicated, nevertheless, valencene was absent or its concentration was too low to accurately measure in the pomelo EOs, which corresponded to our present study. In addition, the significant increase in nootkatone during the storage of Tosa buntan (a variety of pomelo) was not proportional to the change in the level of valencene [40]. Hence, there might be another biosynthetic pathway for nootkatone in pomelo besides that via valencene [41].

Figure 2b,c show that the change in monoterpene hydrocarbon β-phellandrene and monoterpene aldehyde geranial was similar and constant in the first 15 days, decreased to the minimum at 60 days of storage, increased until 90 days and then seemed to be unchanged until 120 days.

β-phellandrene and geranial, the product of the MEP pathway in plant chloroplasts, where β-phellandrene is directly synthesized from geranyl pyrophosphate by the chloroplast-localized β-phellandrene synthase enzyme [42] and the monoterpene aldehyde geranial (in the geraniol degradation pathway), was produced under the function of alcohol dehydrogenase using the corresponding alcohols as substrates [34].

Limonene, the product of the MEP pathway in plant chloroplasts, was the most abundant monoterpene hydrocarbon component in the peel, compatible with previous studies [23,24,25,40]. Figure 2d shows that in the first 15 days, the content of limonene seemed to be constant, then increased from day 15 of storage to 60 days (~8 weeks) of storage and decreased to 90 days (~12 weeks) and after that, seemed to be constant until 120 days. The increasing of limonene at the beginning of storage was similar to the result of other research, e.g., the 7 weeks storage of grapefruit at 2 °C [37,43], the 6 weeks storage of mandarin at 5 °C [37,44] and the 3 weeks storage of lemon at 10 °C [33]. The limonene content in fruit peel was increased. It is due to cold-induced responses [37,43,44]. The storage time of mandarin varieties was found to greatly alter their volatile profiles [45]. There was no storage study after 8 weeks; however, the decreasing of limonene after ~8 weeks to ~12 weeks might be due to more or less higher peel damage leading to more emission of limonene to the atmosphere [43], resulting in low content in the peel and after that, remaining constant.

Figure 2b,c show the same trend in change but is opposite to Figure 2d, which might be due to the same biosynthesis pathway being used and the presence of and change in different substrates. However, there was no significant difference in β-phellandrene, geranial and limonene concentrations between the first 15 days and after 90 days of storage, which might be due that, on those dates, the physical and biochemical properties of the peel were not changed and hence the substrates also were not changed.

**Figure 3:**
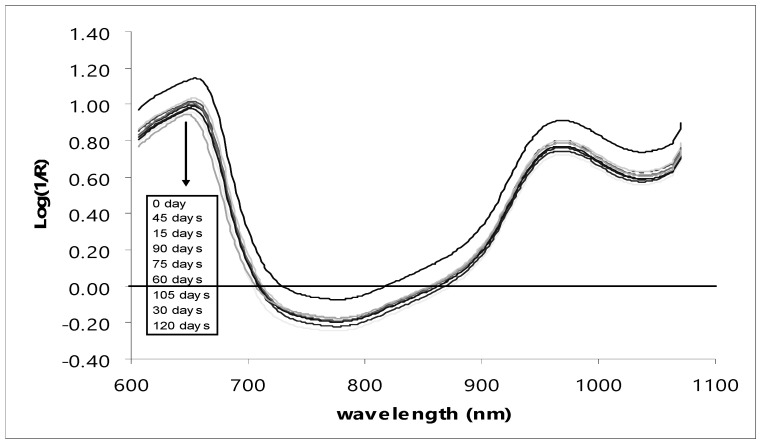
Average original VIS/NIR spectra of intact pomelo at different storage durations.

**Figure 4:**
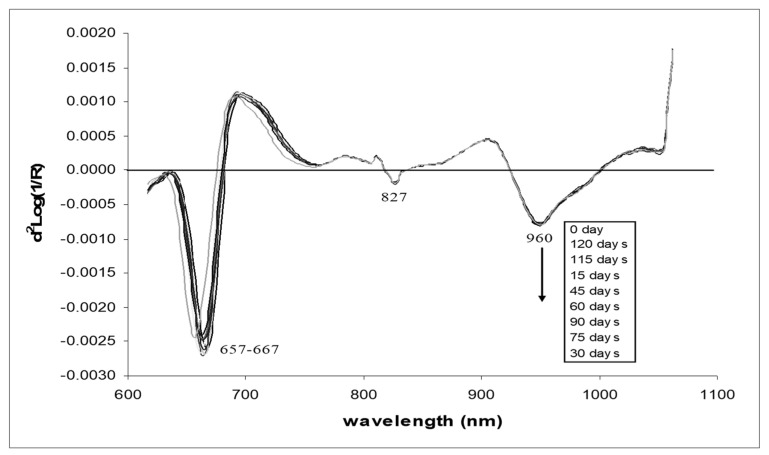
Average second derivative VIS/NIR spectra of intact pomelo at different storage durations.

Figure 3 shows the average original spectra of the pomelos at nine different storage durations. There were significant differences in the optical characteristics of the skin of the pomelos at different storage durations. However, between 15 and 120 days, the spectra were closer to each other, especially when compared with the results obtained on day 0. Figure 4 shows the average second derivative spectra obtained via the Savitzky–Golay method using an 11-points derivative segment with a polynomial order of two for pomelo fruits at nine different storage durations. The average original VIS/NIR absorbance spectra of pomelo showed a similar profile, with absorption maxima at 657–667 and 960 nm. The 960 nm absorbance bands correspond to water bands [46]. The regions around 680 nm are from chlorophyll pigments, which reflect the overall color characteristics of the fruit [47,48]. There was also a small peak observed at 827 nm, which was the shifted peak of 830 nm indicated by Workman and Weyer [49] as the absorbance peak of aliphatic hydrocarbon. Aliphatic hydrocarbon was in the EOs of the citrus peel [50,51].

**Figure 5:**
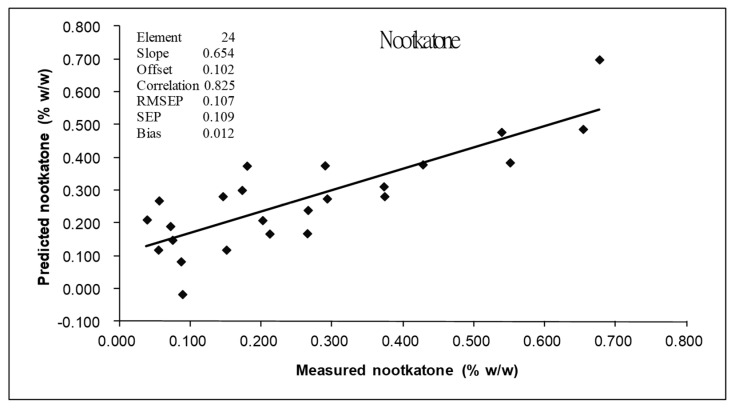
Comparison of measured and predicted values obtained using the PLS calibration equations for nootkatone. RMSEP: root means square error of prediction; SEP: standard errors of prediction; Bias: mean of error.

**Figure 6:**
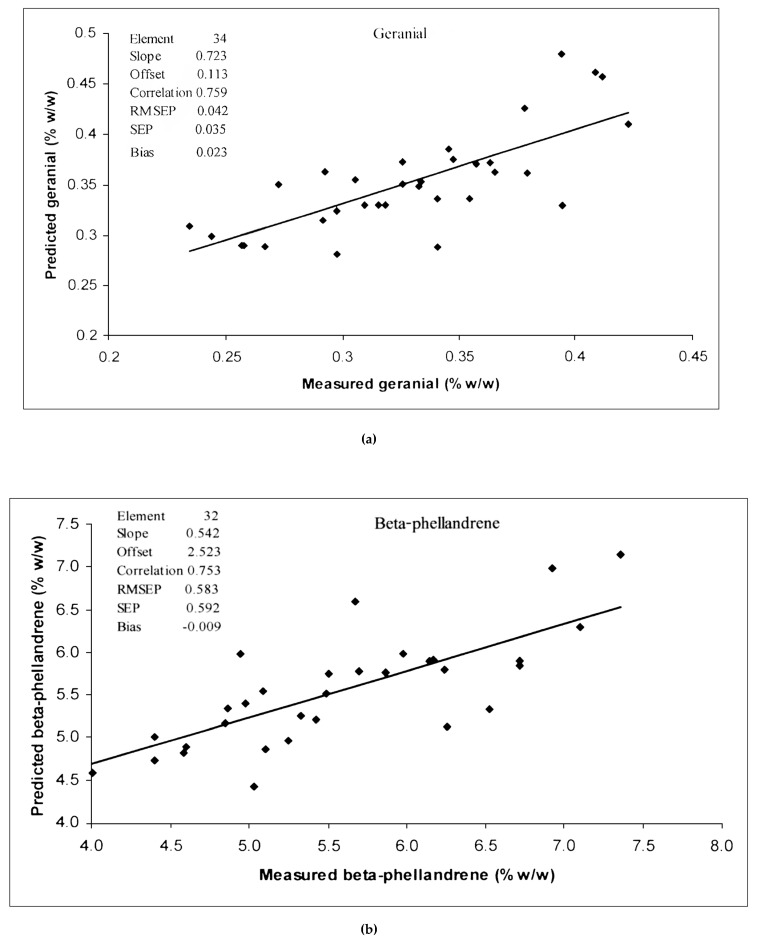

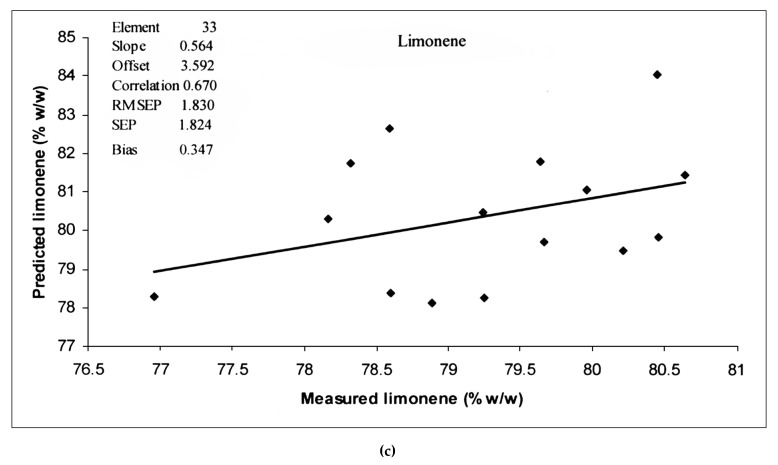
Comparison of measured and predicted values obtained using the PLS calibration equations for (**a**) geranial, (**b**) β-phellandrene model and (**c**) limonene. RMSEP: root means square error of prediction; SEP: standard errors of prediction; Bias: mean of error.

Table 2 shows the statistical values associated with the peel EO components for the calibration and prediction sets. Table 3 shows the results obtained from the PLS regression analysis for each parameter of peel EO obtained from the pomelo fruit (cv Kao Nam Pueng). Only the most predictable models using the best pretreated spectra are shown. Among the constituents analyzed by GC-MS, nootkatone, where the model was developed from the full MSC, had the highest correlation coefficient value (r = 0.82) with a standard error of prediction (SEP) of 0.11%, bias of 0.01% and RPD of 1.7 (Figure 5). The model of geranial, with full MSC had r, SEP, bias and RPD values of 0.76, 0.03%, 0.02%, and 1.5, respectively (Figure 6a), while the model of β-phellandrene, with second derivative pretreatment, had values of 0.75, 0.59%, −0.01% and 1.5, respectively (Figure 6b). The model generated for limonene displayed the lowest accuracy of all, with r, SEP, bias and RPD values of 0.67, 1.82%, 0.35% and 1.3, respectively (Figure 6c). Williams’ guideline [32] indicated that an RPD of < or = 1.9 means that the model was very poor; however, the r of 0.81–0.90 implied the model was applicable for rough screening and approximate calibration, 0.71–0.80 implied the model was for very rough to rough screening and 0.51–0.70 indicated the model was poor and could not be recommend to be used. Therefore, we followed the guideline of r for our model interpretation.

The r values for the prediction set were 0.82, 0.75, 0.76 and 0.67 for the model of nootkatone, β-phellandrene, geranial and limonene. Using Williams, Manley and Antoniszyn [32], the appropriate application of nootkatone, β-phellandrene and geranial models were for screening, approximate calibration (r = 0.81–0.90) and very rough to rough screening (r = 0.71–0.80). But for the limonene model, there was poor correlation (r = 0.51–0.70) and had to be researched for a rationale. It was noticed that the ratio of SEP to SEC of every model was close to 1 (1.0, 1.1, 1.0 and 1.0, respectively), which indicated the calibration sample set and prediction sample set had a similar distribution.

**Figure 7:**
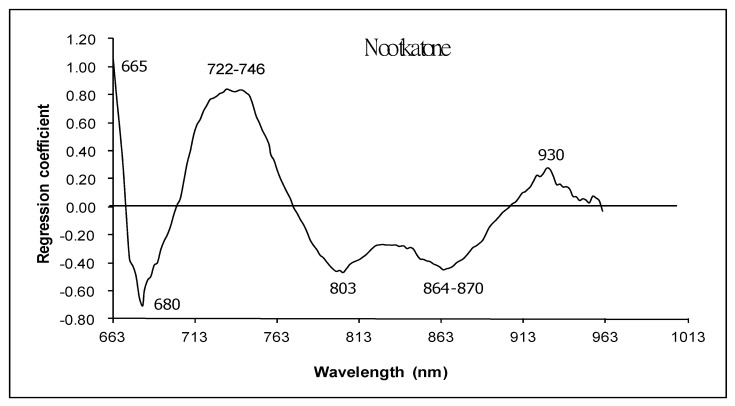
Regression coefficient plot of calibration model for nootkatone obtained from pomelo peel.

Figure 7 shows the regression coefficient plot for the calibration model of nootkatone. It could be seen that at a wavelength of 680 nm, the regression coefficient was fairly high. As mentioned before, 680 nm corresponds to the chlorophyll absorbance band [47,48,52]. This indicated that the change in color of skin related to the nootkatone content. There was also a peak at 930 nm, which is the oil absorption band [46]. Osborne and Fearn [53] also reported that 928 nm is the absorbance band of oil. There were also important wavelengths identified by the model as having large regression coefficients including 655, 722–746, 803 and 827 (aliphatic hydrocarbon absorption [49] and 864–870 nm).

**Figure 8:**
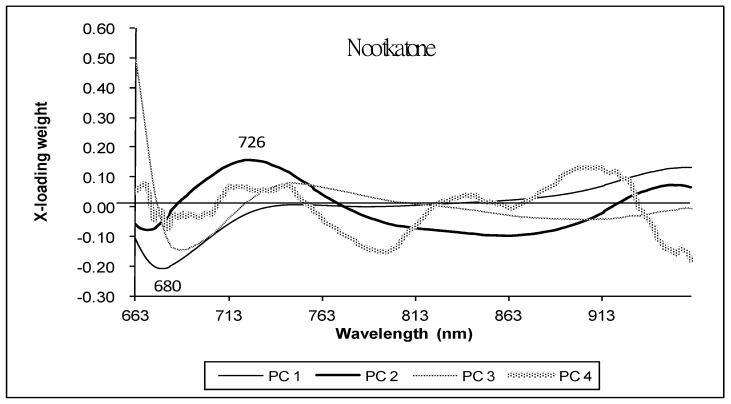
X-loading plot obtained from the PLS calibration model for nootkatone obtained from pomelo peel.

Figure 8 shows the X-loading plot of the calibration model for nootkatone. Similar to the regression coefficient, if the X-loading is high at a specific wavelength, the wavelength has a high effect on the model to predict the response variable, in this case nootkatone. Principal component (PC) 1, PC2, PC3 and PC4 explained 98, 1, 1 and 0% of the variation in the data, respectively. The X-loading weight of PC1 shows that most of the wavelength affected the model, especially 680 nm, which was the chlorophyl absorption peak, except 779–797 nm. In the case of PC2, only 684, 775 and 921 nm had no effect on the model, but they were also shown in PC1, PC3 and PC4. The X-loading weight of PC3 at 663 nm was high. In the case of PC4, the wavelengths 673, 706, 760, 825, 866 and 935 nm had no effect on the model.

**Figure 9:**
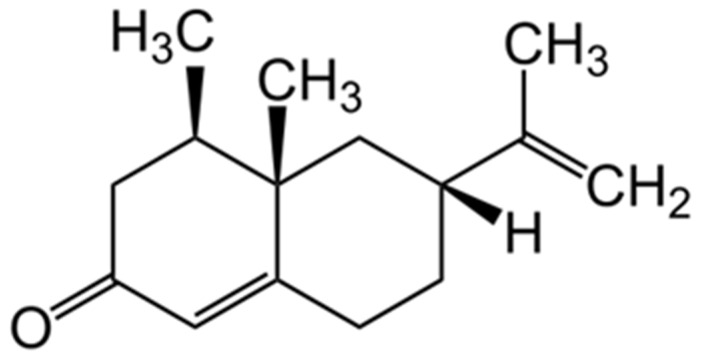
Chemical structure of nootkatone.

Both the regression coefficient plot and X-loading plot could be used to indicate the effect on the model on a molecular basis. Figure 9 shows the chemical structure of nootkatone. There are also CH_3_, CH_2_, C=O and =CH_2_ in the structure. In the regression coefficient plot (Figure 6), the highest peak was found at 722–746 nm. Osborne and Fearn [53] also reported that the absorbance bands of CH_3_ and CH_2_ were at 740 nm and 746 nm, respectively, due to the vibration of C-H stretch occurring as a fourth overtone. However, the C=O and =CH_2_ vibration occur as a second and first overtone, respectively, with the absorbance bands at 1950 nm and 1620 nm [53], respectively. These are not in the range assessed in this study. By considering this fact, it could be the reason why nootkatone could be measured by VIS/NIR spectroscopy in the range of 663–961 nm.

**Figure 10:**
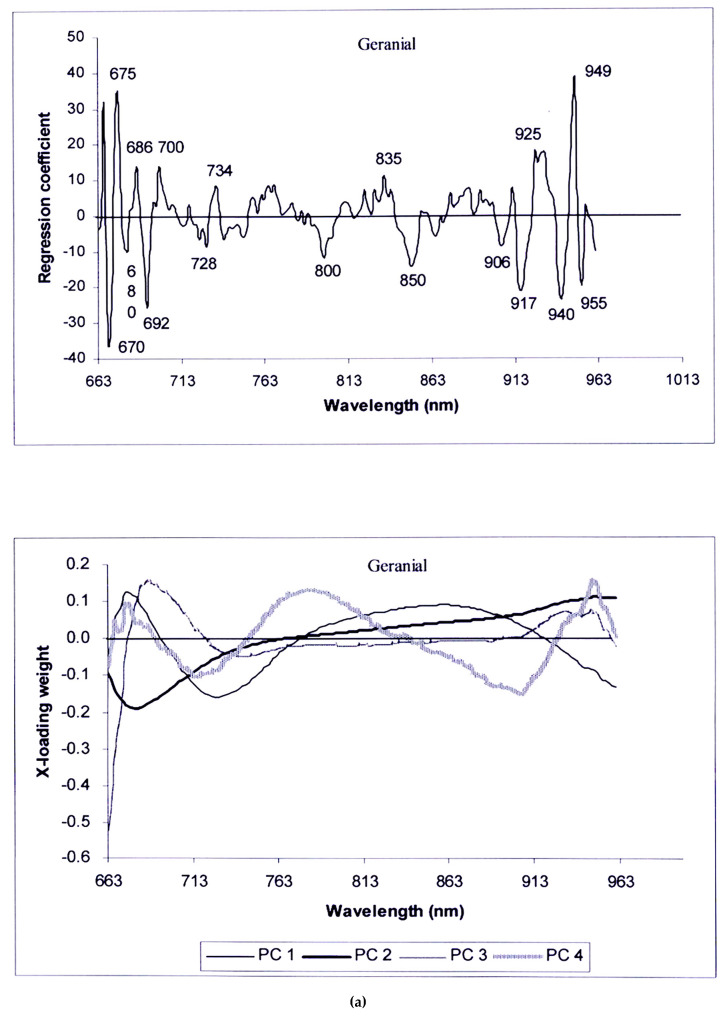

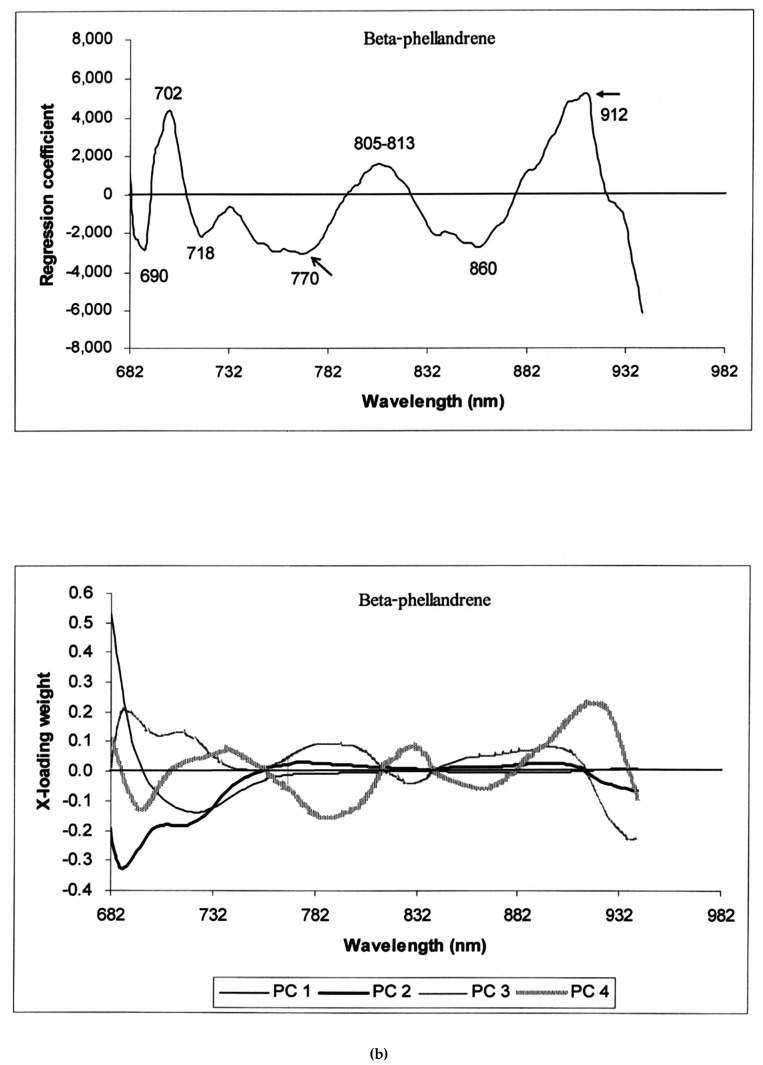

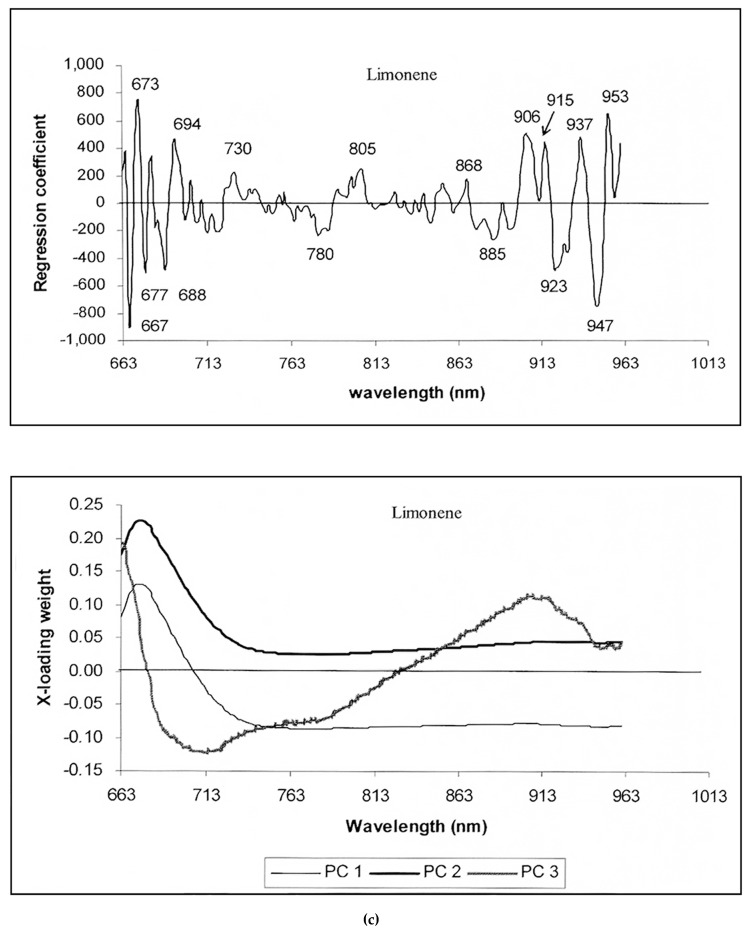
The regression coefficient plot and X-loading plot of (**a**) geranial model; (**b**) β-phellandrene model; (**c**) limonene model.

Figure 10a shows the regression coefficient plot and X-loading plot of geranial, Figure 10b shows those of β-phellandrene, and Figure 10c shows those of the limonene. The corresponding vibration bonds to the high peaks in the regression coefficient plots and X-loading plots, which related to and influence on the prediction of corresponding EO constituents, are described further.

From Figure 10a, the highest peaks in the regression coefficient plot of the geranial model were at 670, 675 and ~950 nm. This corresponded with chlorophyll-a, where its peak absorbance was within 660 nm to 670 nm, which showed a correlation with oil palm fresh fruit free fatty acid, although it is not significant [54]. It was confirmed by the high peak at 680 nm, which is the chlorophyll absorption vibration [47,48,52]. Williams, Manley and Antoniszyn [32] indicated that 964 nm is the peak of water in biological materials; however, there may be differences among instruments and the exact position of these bands might not be the same when using different NIR instruments and might differ by 1–5 nm. Moreover, the positions of the absorption bands in different materials were affected by interactions among the constituents and by temperature too. Regions 950 to 975 nm is associated with water [55]. Therefore, the highest peak at ~950 nm and high peak at 955 nm indicated the effect of water vibration on the prediction of geranial. Hernández-Sánchez and Gómez-Del-Campo indicated that the structure and the distribution of the oil content and the water content within olive cells and tissue, as well as their relative proportions, affected the spectral response. Water is the most important component of fresh olive fruit (48–66%fresh matter) and oil is the second most important component (11–25% fresh matter), hence the absorbance of water would reveal information on the oil content [56]. In case of pomelo peel, the main components of wet peel, like other citrus fruits, include high water content (77% to 80% db) [57,58], lipids (mainly D-limonene) and other compounds [57]. Therefore, the vibration of water could reveal the information of oil, i.e., in this case, EO components. Also the high peak at 925 nm was by the absorption of oil, and Williams, Manley and Antoniszyn [32] indicated that 928 nm is the peak of oil in biological materials.

The PC1, PC2, PC3 and PC4 covered the variance of 62, 37, 1 and 0%, respectively. The X-loading weight of PC1 shows the vibration of most wavelengths except 665, 694, 779 and 919 nm have an influence on the prediction of the model, which is similar to the X-loading weight of PC2, where except 767 nm, its influence shows in PC1, PC3 and PC4. 

The vibration at 675, 720 and 896 nm in the X-loading weight of PC3 has no effect, but its influence shows in PC1, PC2 and PC4; however, the X-loading weight of PC4 indicated an important wavelength of 950 nm.

From Figure 10b, the highest peaks in regression coefficient plot of β-phellandrene model were at 702 and 912 nm and high peak in the range of 805–813 nm. Williams, Manley and Antoniszyn [32] indicated that 708, 808 and 908 nm are the peaks of protein in biological materials. Therefore, the prediction of β-phellandrene by VIS/NIRS might be related to the protein content in pomelo peel. This corresponded to Choung et al. [59], which indicated that the prediction of protein content in canola or soybean might use a wavelength associated with oil, due to a strong negative relationship (r = −0.80 to −0.90) between the oil and protein contents of these materials. Hence, it was vice versa in our case of the prediction of β-phellandrene.

The PC1, PC2, PC3 and PC4 covered the variance of spectral variables of 95, 4, 1 and 0%, respectively. For PC1 to PC4, the wavelength range of 682–710 nm had higher X-loading than most of the other ranges.

From Figure 10c, the highest peaks in the regression coefficient plot of limonene model were at 667 and 673 nm, indicating the effect of chlorophyll-a on the EO prediction, and at 947 and 953 nm, indicating the effect of water on the prediction.

The PC1, PC2 and PC3 covered the variance in the spectral variables of 63, 36 and 0%, respectively. The X-loading plot of PC1, PC2 and PC3 of limonene model shows the highest peaks in chlorophyll-a bands at the range of 660 nm to 670 nm [54] and of PC1 and PC2 at 680 nm in chlorophyll [47,48,52]. The high peak of PC3 in the 900–920 nm range confirms that the relevant spectral information is obtained primarily from a carbohydrate/lipid CH absorbance band [60].

It is worthy to prove whether nonlinear modeling performed better than linear model of classical PLS regression. Therefore, the support vector machine (SVM) and artificial neural network (ANN), which were the algorithms for nonlinear modeling, were calculated. It appears that both do not significantly improve prediction performance (Table 4). However, for SVM, the prediction r was lower than calibration, which was reasonable. But for ANN, the prediction r is much lower or even sometimes higher than the calibration result, so it was overfitting.

The SVM was performed on a calibration set with a fine-tuning hyperparameter, including kernel function and degree of polynomial function. The four popular kernel functions, including linear, polynomial, radial basis function (RBF) and sigmoid, were applied and the degree of polynomial function for fine-tuning was about 2–6. The optimal hyperparameter was found by the GridSearchCV command of the Scikit-learn module with the 5-fold cross-validation based on the maximum of R. The SVM model provided r values of 0.62, 0.68, Na and Na for nootkatone, geranial, β-phellandrene and limonene, respectively (Table 4). Na is not applicable.

The ANN model includes a specified number of hidden layers (1, 2 or 3 layers) and a varying number of nodes in each hidden layer (starting from 10 and increasing in increments of 10 until reaching 100). Each model with selected parameters was generated 20 times to identify the best model, which was selected based on the highest r value and the r that was closest to R value. The ANN model obtained r values of 0.79, 0.43, 0.27 and 0.51 for nootkatone, geranial, β-phellandrene and limonene, respectively (Table 5).

In comparison, the PLS model achieved R values of 0.82, 0.75, 0.76 and 0.67 for the same compounds. The performance values of the SVM and ANN model did not show improvement, and overfitting was observed, indicating that the data of the EOs were more linear than nonlinear.

## 4. Discussion

The present work investigated the change in pomelo (cv Kao Nam Pueng) peel EO constituents including nootkatone, β-phellandrene, geranial and limonene during long-term (4 months) cold storage and the feasibility of the constituent measurement by VIS/NIR spectroscopy, which was in the wavelength range of 600–1100 nm. This is the first report of the use of NIR spectroscopy on intact fruit measurement for its peel EO constituents during cold storage.

In the past, to our knowledge, VIS/NIR (600–1100 nm) spectroscopy had not been used for the determination of EO yield in citrus peel and other oil crop. But in the longer wavelength range, hand-held NIR (1000–1800 nm) and FT-NIR (1000–2500 nm) was used for the determination of EO content of oregano and obtained an R^2^ of 0.58 and 0.91, respectively [61]. This indicated that the wavelength between 1800 and 2500 nm was effective in the evaluation. This was confirmed by the investigation of potential of NIR hyperspectral imaging in the spectral range of 1000–2500 nm, which was used for the prediction of oil concentration in five peanut cultivars and obtained an R^2^ of 0.945 [38]. The range of 935 to 1720 nm was used for the determination of EO content in dried spearmint (*Mentha spicata* L.) (0.2 to 2.6%), which obtained an R^2^ of prediction of 0.863 [62]. These might be an indication of why VIS/NIR could not obtain high performance due to the less informative variables in the range of shortwave NIR for oil evaluation.

There was only a report [13] for the EO components (e.g., limonene, γ-terpinene, sabinene) and some chemical–physical parameters in various intact citrus EOs including grapefruit, orange, mandarin, lemon and lime EOs, which were evaluated using NIR (1100–2500 nm) and obtained an R^2^ of >0.95 for components with a concentration >1.5%.

A study by Sirisomboon and Duangchange [27], which was the first report on using NIR spectroscopy to determine the EO components by scanning the peel of intact fruits during maturity change, where VIS/NIR spectrometer was used and the pomelo (cv Kao Nam Pueng) fruits from 190 to 220 days after flower blooming were the samples, concluded that the EO components analyzed by GC-MS including limonene (78.33–84.93%), β-phellandrene (4.16–6.22%) and α-pinene (1.33–1.85%) showed that β-phellandrene provided the highest value of r between predicting values and reference values (r = 0.89) with an SEP of 0.39% and bias of 0.39%.

Essential oils were extracted from the peel of four citrus: bitter orange (*Citrus aurantium*), lemon (*Citrus limon*), orange maltaise (*Citrus sinensis*) and mandarin (*Citrus reticulate*), which varied during ripening from 0.46 to 2.70% [63]. In our case of pomelo (*Citrus maxima*) during cold storage, the limonene content was the maximum (76.02–87.82% in the oil) and nootkatone content was the minimum (0.02–0.98% in the oil). Though it was the minimum, the nootkatone model, where the model was developed from the full MSC, had the highest r = 0.82 with an SEP of 0.11% and bias of 0.01%. The model generated for limonene, the maximum content, displayed the lowest performance of all, with r, SEP and bias values of 0.67, 1.82% and 0.35%, respectively.

However, the combination and overtone vibration in the NIR region that are caused by O-H, C-H and N-H bonds result in broad overlapping spectra, and the sensitivity of these techniques is limited to approximately 0.1% for trace components [64]. The explanation as to why the low quantity of nootkatone constituents, which was less than 0.1%, could be evaluated by NIR spectroscopy is in the discussion in the end of Results section of this manuscript; the strong vibration of every carbon and hydrogen bond but excepted C=O and =CH_2_ in the chemical structure of nootkatone in the EO were effectively influenced by the prediction of the model illustrated by the regression coefficient plot and X-loading plot, where the chemical phenomena and effect could be retrieved. There were the same occurrences in the following: the determination of gamma oryzanol and gamma aminobutyric acid (GABA) in germinated brown rice, where the content of gamma oryzanol and GABA was very low, at 6.06 × 10^−4^–3.27 × 10^−3^ mg/100 g dry matter and 1.24–6.38 mg/100 g dry matter, respectively, and the R^2^ of the prediction model developed using FT-NIR spectrometer (12,500–4000 cm^−1^ or 800–2500 nm) was 0.934 [65] and 0.981 [66], respectively, and the determination of deoxynivalenol (concentration range of 0–90 mg/kg) in whole wheat grain by means of FT-NIR spectrometer (10,000–4000 cm^−1^ or 1000–2500 nm), where an R^2^ of 0.94 was obtained [67]. These results, along with our results, confirm that no matter how small the amount of the constituents, as long as the NIR absorber bonds in the sample related to the constituents strongly vibrate, the constituents can be predicted by NIR spectroscopy. In addition, the increasing quantity of nootkatone (Table 1 and Figure 2) occurring simultaneously with the physicochemical change in the pomelo peel during storage studied by our group [68] corresponded to the change in the VIS/NIR spectra, while the quantity of limonene though the highest percentage in the peel fluctuated (Table 1 and Figure 2) during the physicochemical change in pomelo peel during storage [56] was not corresponded to the change in the VIS/NIR spectra of the pomelo. This might be another reason why the nootkatone model was the best in this study. In addition, a review of the literature on the biosynthesis pathway and the production of nootkatone, geranial, β-phellandrene, and limonene in the Results section described why the EO constituents generated and changed during cold storage.

## 5. Conclusions

The PLS model using visible and near-infrared spectroscopy (VIS/NIRS) in this study (r, SEP and bias of 0.82, 0.11% and 0.01%, respectively) could be used for the screening and estimation of nootkatone, an EO constituent found in pomelo peel. Though the quantity of nootkatone constituents in pomelo peel was low, the strong vibration of every carbon and hydrogen bond but excepted C=O and =CH_2_ in the chemical structure of nootkatone in the EO effectively influenced the prediction of the model illustrated by the regression coefficient plot and X-loading plot, which shows a breakthrough in the limitations of NIR technology. Therefore, it is suggested that VIS/NIR spectroscopy technique, as described here, has the potential to replace existing traditional methods, which consume much more time and chemicals, but can only be applied to the screening and approximate calibrations of nootkatone in pomelo peel. The change in the amount of major EO constituents (nootkatone, β-phellandrene, geranial and limonene) during storage of 4 months was also reported. The generation of and change in the EOs were explained by the biosynthesis pathway specifically. This technique and information could be potentially applied to the extraction process in the industrial production of EO and in academic research institutes, which conduct breeding research and product development research. The chemical bond vibrations affecting the prediction of EO constituents was comprehensively explained by the regression coefficient plots and X-loading plots. Further research is recommended to use longwave NIR (1100–2500 nm) on intact fruit for modeling and to input more stored samples of different cold storage duration to make a robust model and with more varieties and locations to make global models.

## Figures and Tables

**Table 1:** Concentration of the major essential oil components from pomelo peel (cv Kao Nam Pueng) at different storage durations by GC-MS.

Storage Days	Mean Concentration (% *w*/*w*)			
	Nootkatone	β-Phellandrene	Geranial	Limonene
0	Not detectable	6.16 ± 0.56 ^a^	0.39 ± 0.06 ^a^	79.95 ± 1.63 ^cd^
15	Not detectable	6.22 ± 0.62 ^a^	0.40 ± 0.05 ^a^	80.37 ± 1.93 ^cd^
30	0.06 ± 0.02 ^e^	5.27 ± 0.54 ^bc^	0.33 ± 0.04 ^c^	83.07 ± 1.72 ^b^
45	0.08 ± 0.05 ^e^	4.85 ± 0.66 ^cd^	0.31 ± 0.04 ^cd^	84.23 ± 1.5 ^ab^
60	0.13 ± 0.07 ^de^	4.64 ± 0.59^d^	0.27 ± 0.03 ^d^	84.61 ± 1.42 ^a^
75	0.22 ± 0.12 ^cd^	5.00 ± 0.73 ^cd^	0.31 ± 0.04 ^cd^	83.89 ± 1.98 ^ab^
90	0.33 ± 0.16 ^c^	6.32 ± 1.02 ^a^	0.33 ± 0.05 ^bc^	79.54 ± 2.36 ^d^
105	0.47 ± 0.30 ^b^	5.70 ± 0.98 ^ab^	0.31 ± 0.07 ^cd^	81.13 ± 1.90 ^c^
120	0.63 ± 0.22 ^a^	6.15 ± 0.9 ^a^	0.37 ± 0.07 ^ab^	79.17 ± 2.09 ^d^

Different letters in the same column represent significant difference at *p* ≤ 0.05 by Duncan’s multiple range tests.

**Table 2:** Basic statistics of each oil constituent from pomelo (cv Kao Nam Pueng) of the calibration and prediction sets.

Parameters	Calibration Set				Prediction Set			
	n	Range (%)	Mean (%)	SD (%)	n	Range (%)	Mean (%)	SD (%)
Nootkatone	71	0.02–0.98	0.26	0.22	24	0.04–0.68	0.26	0.19
β-phellandrene	87	3.67–7.54	5.57	0.92	32	3.99–7.36	5.54	0.90
Geranial	90	0.16–0.51	0.33	0.06	34	0.23–0.42	0.32	0.05
Limonene	93	76.02–87.82	81.79	2.72	33	78.17–85.94	81.71	2.39

n is number of fruits. SD is standard deviation.

**Table 3:** Statistical results obtained from PLS regression models for the calibration and prediction sets for each oil constituent from pomelo (cv Kao Nam Pueng).

Parameters	Pretreatment	PCs	Calibration			Prediction			
			r	SEC (%)	Bias (%)	r	SEP (%)	Bias (%)	RPD
**Nootkatone**	**Full MSC**	**4**	**0.85**	**0.11**	**1.49 × 10−8**	**0.82**	**0.11**	**0.01**	**1.7**
β-phellandrene	2nd derivative	5	0.73	0.63	−1.01 × 10−7	0.75	0.59	−0.01	1.5
Geranial	Full MSC	12	0.84	0.03	9.14 × 10−7	0.76	0.03	0.02	1.5
Limonene	Raw	10	0.76	1.76	1.48 × 10−5	0.67	1.82	0.35	1.3

PCs are number of latent variables or (PLS) principal components. r is the correlation coefficient. SEC is standard error of calibration. SEP is standard error of prediction.

**Table 4:** The performance of SVM model for EOs of pomelo peel using VIS/NIR spectroscopy.

Parameters	Pretreatment	Kernel	Degree	Calibration		Prediction		
				R	SECV	r	SEP	Bias
nootkatone	D2	Poly	3	0.86	0.092	0.62	0.196	−0.009
geranial	D2	Poly	3	0.88	0.018	0.68	0.052	−0.002
β-phellandrene				Na		Na		
limonene				Na		Na		

**Table 5:** The performance of ANN (average spectra) model for EOs of pomelo peel using VIS/NIR spectroscopy.

Parameters	Pretreatment	Hidden Layer	Calibration		Prediction		
			R	SECV	r	SEP	Bias
nootkatone	D1	70 10 10	0.98	0.05	0.79	0.16	−0.02
geranial	D2	60 10	1.00	0.00	0.43	0.06	0.01
β-phellandrene	Mean normalization	40 10	0.62	0.77	0.27	0.86	−0.19
limonene	Mean centering	80 10	0.29	3.76	0.51	1.98	0.36

## Data Availability

The original contributions presented in the study are included in the article, further inquiries can be directed to the first author and corresponding author.
